# Bundle-specific tractography approach for identifying white matter microstructural changes following traumatic brain injury in rats: An EpiBioS4Rx study

**DOI:** 10.1162/imag_a_00471

**Published:** 2025-02-18

**Authors:** Akul Sharma, Ryan Cabeen, Neil G. Harris, David K. Wright, Jussi Tohka, Olli Grohn, Alexis Bennett, Celina Alba, Sweta Bhagavatula, Rachael Garner, Tuba Asifriyaz, Xavier Ekolle Ndode-Ekane, Pedro Andrade, Riikka Immonen, Eppu Manninen, Noora Puhakka, Mette Heiskanen, Idrish Ali, Nigel C. Jones, Sandy R. Shultz, Pablo M. Casillas-Espinosa, Matthew R. Hudson, Juliana Silva, Glenn Yamakawa, Emma L. Braine, Gregory D. Smith, Cesar E. Santana-Gomez, Richard J. Staba, Terence J. O’Brien, Asla Pitkanen, Dominique Duncan

**Affiliations:** Laboratory of Neuro Imaging, USC Stevens Neuroimaging and Informatics Institute, Keck School of Medicine of USC, University of Southern California, Los Angeles, CA, United States; UCLA Brain Injury Research Center, Department of Neurosurgery, Geffen Medical School, David Geffen School of Medicine at UCLA, Los Angeles, CA, United States; Intellectual Development and Disabilities Research Center, University of California at Los Angeles, Los Angeles, CA, United States; The Department of Neuroscience, The School of Translational Medicine and Alfred Health, Monash University, Melbourne, Australia; A.I. Virtanen Institute for Molecular Sciences, University of Eastern Finland, Kuopio, Finland; Department of Neurology, David Geffen School of Medicine at UCLA, Los Angeles, CA, United States

**Keywords:** magnetic resonance imaging, traumatic brain injury, diffusion tensor imaging, tractography, fluid-percussion injury, biomarkers

## Abstract

Traumatic brain injury (TBI) presents a major global health concern, characterized by a variety of negative long-term neurological outcomes. Current diagnostic tools lack the sensitivity to fully capture the complex pathophysiology of TBI and predict long-term consequences, underscoring the need for robust methods for biomarker detection. This study, conducted within the multicenter Epilepsy Bioinformatics Study for Antiepileptogenic Therapy (EpiBioS4Rx) framework, used a standardized lateral fluid-percussion injury (FPI) model to produce TBI in the left hemisphere of adult male Sprague-Dawley rats across three sites: University of Eastern Finland, Monash University, and the University of California, Los Angeles. This study utilized a novel kernel regression method for improved estimation of fiber orientations and streamline tractography to derive diffusion tensor imaging (DTI) metrics of 36 white matter tracts which were used as features to classify TBI versus sham-operated rodents scanned at 2 days (30 sham, 87 TBI), 9 days (29 sham, 84 TBI), 1 month (28 sham, 81 TBI), and 5 months (25 sham, 65 TBI) post-injury using elastic net regression regularization. A mean area under the curve (AUC) of 0.92 was achieved in correctly classifying the TBI rats in a leave-one-out cross-validation (LOOCV) framework. The results revealed delayed, region-specific effects on the microstructure of the left fimbria and left thalamic subcortical projections at 5 months following TBI. By integrating multi-compartment modeling, tractography, and harmonization, this study advances our understanding of the temporal evolution of TBI pathogenesis, paving the way for development of translational prognostic biomarkers for the risk of post-traumatic epilepsy (PTE).

## 1 Introduction

Traumatic brain injury (TBI) is a leading cause of disability and death worldwide, resulting in long-term cognitive, psychological, and neurological deficits, and the development of post-traumatic epilepsy (PTE) (Hyder et al., 2007;Jha & Ghewade, 2022). However, our understanding of its pathophysiology and long-term consequences remains limited primarily due to its heterogeneous nature and the lack of effective clinical interventions. One of the most significant challenges in addressing TBI is the absence of reliable biomarkers that can facilitate early diagnosis, predict long-term outcomes, and guide treatment decisions (Saatman et al., 2008). As a result, clinical management is often challenging, and the ability to predict which patients will develop lasting deficits remains limited (Borg et al., 2004;Ruff et al., 2009). Given the high prevalence of TBI and its long-term consequences, there is a critical need to identify biomarkers that can improve diagnostic accuracy and guide appropriate interventions and treatment options (Thompson et al., 2014).

Diffusion magnetic resonance imaging (dMRI), particularly diffusion tensor imaging (DTI), has emerged as a valuable tool for assessing white matter (WM) microstructure in TBI (Le Bihan et al., 2001). DTI-derived metrics such as axial diffusivity (AD), radial diffusivity (RD), mean diffusivity (MD), and fractional anisotropy (FA) provide insights into axonal integrity, myelination, and overall brain connectivity (Budde et al., 2009;Huisman et al., 2004;Johnson et al., 2013). Lateral fluid-percussion injury (LFPI) is currently the most widely used preclinical model of human TBI and has been established as a valid and reliable model to study the pathophysiology of human TBI (McIntosh et al., 1989). It has been used primarily to identify TBI-induced cellular and molecular changes including white and gray matter damage (Thompson et al., 2005). Studies using LFPI models and DTI have shown that TBI induces significant changes in white matter tracts, such as the corpus callosum, fimbria, and internal capsule, at chronic post-injury time points (Cabeen et al., 2020;Harris et al., 2016;Johnstone et al., 2015;Laitinen et al., 2015;Quach et al., 2024;Shenton et al., 2012;Wright et al., 2016;Xiong et al., 2014). These microstructural diffusion alterations reflect the chronic pathophysiology of TBI, including axonal degeneration, myelin loss, and structural reorganization (Garner et al., 2019;Pitkänen et al., 2009).

However, conventional diffusion tensor models are constrained by their single-fiber orientation representation and struggle in areas of complex fiber structures, such as regions with crossing fibers (Homos et al., 2017;Tournier et al., 2002). TBI induces chronic microstructural changes which further complicate the fiber geometry (Irimia et al., 2012), leading to errors in tractography and further clinical interpretation (Yeh et al., 2019). To overcome this limitation, we utilize a novel kernel regression framework that leverages a collection of fiber models contained in a volumetric parametric map to estimate fiber orientation mixtures (Cabeen et al., 2016,^2020^;Cabeen & Toga, 2020). This method allows for more accurate modeling of complex anatomical features by incorporating directional measures of divergence and using data-adaptive extensions for model selection and bilateral filtering (Takeda et al., 2007;Taquet et al., 2012).

This study is part of the Epilepsy Bioinformatics Study for Antiepileptogenic Therapy (EpiBioS4Rx) initiative (Vespa et al., 2019). While the larger EpiBioS4Rx project includes a focus on PTE, the current study focuses exclusively on TBI-induced structural changes and does not include seizure-related data. Using a multi-site dataset from University of Eastern Finland (UEF), Monash University, and University of California, Los Angeles (UCLA), we utilized the LFPI model to compare TBI and sham-operated controls. Integrated within a comprehensive computational framework, the kernel regression approach has been successfully applied in our previous EpiBioS4Rx work using a single-site cohort from UEF, where it demonstrated effectiveness in detecting microstructural abnormalities and identifying distinct white matter responses to injury over time (Cabeen et al., 2020).

Our study expands the application of this analysis framework, incorporating a harmonization pipeline into a broader multi-site dataset as part of the EpiBioS4Rx study. We used the LFPI model to induce TBI in adult male rats and compared them with sham-operated controls, assessing white matter using DTI metrics over four post-injury time points. Specifically, we hypothesized that our framework, including cross-site harmonization, could accurately distinguish TBI from sham rats using multi-site data while maintaining biological variability. In this study, we explored TBI-induced white matter structural alterations at 2 days, 9 days, 1 month, and 5 months post-injury. Using elastic net regression (ENR), we aimed to classify TBI versus sham rats and identify the most distinguishing features for classification. Leveraging the extensive multicenter data available from the EpiBioS4Rx study, this approach provides deeper insight into the temporal evolution of TBI pathology and will play a pivotal role in future EpiBioS4Rx initiatives and broader TBI and PTE research (Duncan et al., 2019).

## 2 Methods

### 2.1 EpiBioS4Rx rodent cohort

Three sites, UEF (Finland), Monash University (Australia), and UCLA (USA), from the international multicenter-based project EpiBioS4Rx were involved. Adult male Sprague-Dawley rats were used at all study sites. In UEF and UCLA, rats were purchased from outside vendors (Envigo Laboratories B.V., The Netherlands; Charles River, USA), while in Monash, 75% were purchased from external vendors and 25% were in-house bred. The mean body weight of the rats at the start of the experiment was 354 ± 18 g (UEF), 349 ± 39 g (Monash), and 336 ± 41 g (UCLA). The study design, procedural harmonization, and model production of the EpiBioS4Rx cohort are described in detail byNdode-Ekane et al. (2019,^2024^). The final cohort utilized for this study is shown inTable 1.

**Table 1.** Number of magnetic resonance imaging scans at different time points and in different treatment groups (TBI and sham-operated experimental controls) used for this study.

Time point	Site	Sham (n)	TBI (n)
2 days	UEF	9	25
	Monash	9	29
	UCLA	12	33
**Total**		**30**	**87**
9 days	UEF	9	25
	Monash	8	29
	UCLA	12	30
**Total**		**29**	**84**
1 month	UEF	9	25
	Monash	7	26
	UCLA	12	30
**Total**		**28**	**81**
5 months	UEF	8	24
	Monash	5	12
	UCLA	12	29
**Total**		**25**	**65**

### 2.2 Lateral fluid-percussion injury

Lateral fluid-percussion injury (FPI) was induced according to the protocol described byKharatishvili et al. (2006)under isoflurane anesthesia (Ndode-Ekane et al., 2019;Shultz et al., 2013). The rats were anesthetized with 5% isoflurane and placed in a stereotactic frame. A midline incision was made to expose the skull, and a 5-mm craniotomy was created over the left convexity using a handheld trephine (UEF, UCLA) or a drill (Monash). After the surgery, the rat was disconnected from the stereotactic frame. The duration of the surgery and anesthesia were recorded. The rat was connected to an FPI device after the toe pinch reflex had returned. A severe TBI was induced using a straight tip in the fluid-percussion device (AmScien FP 302, USA). The mean impact pressure across sites was 2.66 atm (Ndode-Ekane et al., 2024). Post-impact apnea duration and seizure-like behavior were recorded.

All surgical procedures were performed by the same person at UEF, and by three persons at Monash and UCLA to ensure consistency. Post-TBI pain alleviation was provided at all sites. At UEF, rats were treated with buprenorphine (Orion Pharma, Finland) as part of their pain management. Monash followed a similar protocol using buprenorphine (Indivior Pty Ltd, Australia). At UCLA, flunixin meglumine (MERK, USA) was used for analgesia. Additionally, UCLA administered Flu-Nix (Flunixin Meglumine, AgriLabs, USA) as another analgesic option. Physiological parameters were monitored for 30 days post-injury and monthly thereafter.

The method included in this manuscript is adapted fromNdode-Ekane et al. (2024), and detailed information on the procedures, sites, and equipment used in the study is provided in SupplementaryTable S1.

### 2.3 Magnetic resonance imaging acquisition

Imaging was performed at 2 days, 9 days, 1 month, and 5 months post-injury (Table 1). Diffusion-weighted imaging (DWI) for DTI and tractography was performed by single-shot spin echo 3D echo-planar imaging (EPI), with diffusion gradient duration (δ) = 4.2 ms, diffusion gradient separation (Δ) = 12 ms, and b-value = 2,800 s/mm^2^in 42 non-collinear directions with 4 non-diffusion-weighted (b0) images, TR 1,000 ms, TE 26 ms, EPI echo spacing 0.269 ms with 250 μm^3^isotropic resolution, field of view 24 × 18 × 12.3 mm^3^, encoding matrix 96 (read) × 54 (PE1) × 49 (PE2), zero-filled to 96 × 72 × 49, spectral width 357 kHz, 1 average, and fat suppression and outer volume saturation (OVS) with 4 saturation bands, resulting in a scan time of 37 min. The scans were acquired using a combination of 4.7T and 9.4T Bruker Biospec for Monash and 7T Bruker BioSpin MRI for UEF and UCLA. Temperature during scans was carefully controlled using a heated water-circulation system and rectal probes to maintain the animals’ body temperature at 37°C, minimizing variability in diffusion measures due to temperature.

The final MRI cohort consisted initially of 36 sham and 87 TBI rats at 2 days. Subsequently by the 5 months time point, the cohort consisted of 25 sham and 65 TBI rats due to mortality, exclusions due to quality control, and technical difficulties (Ndode-Ekane et al., 2024). The number of rats used at each time point for each site is shown inTable 1.

### 2.4 Data processing

DWI images were processed using an established computational framework consisting of several software packages, including FSL (Jenkinson et al., 2012), Quantitative Imaging Toolkit (QIT) (Cabeen et al., 2013), and MRtrix (Tournier et al., 2019). A general overview of this framework is presented inFigure 1. First, Bruker data were converted to NIfTI using Bru2Nii2 (https://github.com/neurolabusc/Bru2Nii), and subject identifiers and volume file names were harmonized (Bhagavatula et al., 2023;Immonen, Smith, et al., 2019). Quality control was performed by visually inspecting mosaic image plots by estimating the global noise characteristics, and by inspecting noise box plots depicting the entire cohort to detect outliers. Next, the DWI images were Multichannel Principal Component Analysis (MP-PCA) denoised (Veraart et al., 2016) and corrected for Gibbs ringing artifacts (Kellner et al., 2016), eddy current artifacts (Andersson et al., 2016), and bias field distortions using the N4 algorithm (Tustison et al., 2010). Skull stripping was performed by applying FSL BET (Smith, 2002) to the average baseline scan. Diffusion tensors were estimated using FSL DTIFIT and multi-tensor models (ball-and-sticks) using FSL BEDPOSTX (Behrens et al., 2007). Deformable registration was performed using the Advanced Normalization Tools (ANTs) software (Avants et al., 2009) through the VolumeRegisterDeformAnts command in QIT to create a population average DTI dataset. A population averaged multi-tensor data were created using the model-based kernel regression framework for fiber orientation mixtures to inspect population averaged maps of DTI parameters, and the fiber orientations and volume fractions.

**Fig. 1.**
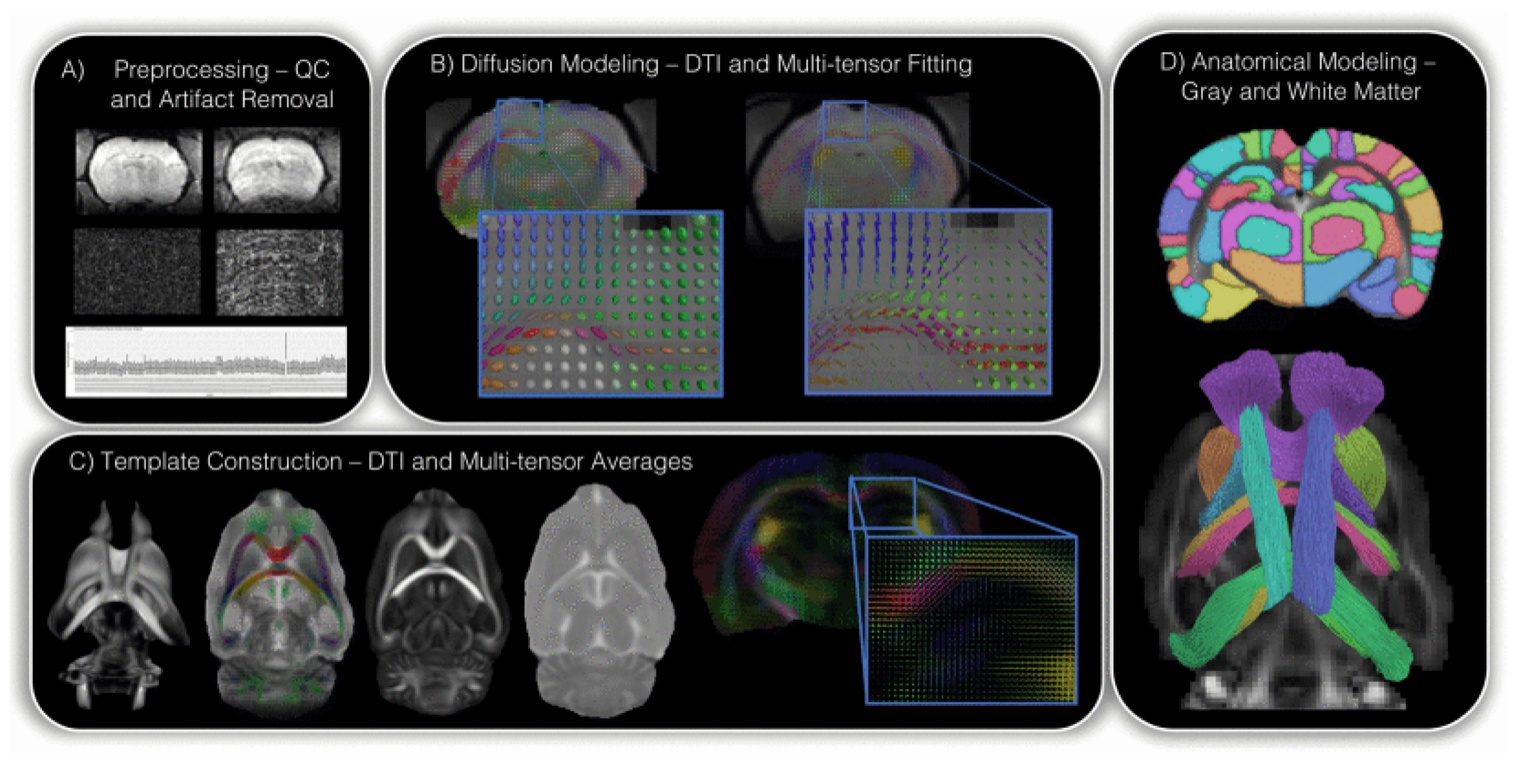
An illustration of the computational diffusion MRI framework, which includes steps for (A) preprocessing, (B) diffusion modeling, (C) DTI and multi-tensor construction, and (D) anatomical modeling, which is done at group and individual levels. Adapted fromCabeen et al. (2020). A computational diffusion MRI framework for biomarker discovery in a rat model of post-traumatic epileptogenesis. In*2020 IEEE 17th International Symposium on Biomedical Imaging (ISBI)*(pp. 1013–1017). IEEE.

### 2.5 Bundle-specific tractography

For tractography analysis, a bundle-specific approach was utilized using an adapted version of the tractography analysis pipeline from our previous framework (Cabeen et al., 2020). First, a prototypical example of each bundle was extracted from the average multi-tensor sham rat data using manually guided seeding, inclusion, and exclusion (Cabeen et al., 2013,^2016^). For each bundle, we computed tract orientation maps (TOM) and inclusion masks for the bundle starting and ending points. TOMs were used as priors in subject-specific bundle reconstruction using a reinforcement tractography method (Cabeen et al., 2020). The TOMs were deformed to subject native space of each scan, and we selected only the closest tensor compartment to the TOM from the subject multi-tensor data based on angular deviation. Streamline tractography was performed with the deformed inclusion mask. Mean FA, MD, RD, and AD were calculated for each of 47 fiber bundles. The list of fiber bundles and their corresponding abbreviations are given inTable 2.

**Table 2.** List of white matter fiber bundles for which the mean FA, MD, RD, AD values were summarized.

Code	Fiber bundle
amyhypdor *	amygdala-hypothalamus dorsal connections
amyhypven *	amygdala-hypothalamus ventral connections
ant comm	anterior commissure
atr	anterior thalamic radiation
cc ant	corpus callosum anterior portion
cc mid	corpus callosum middle portion
cc post	corpus callosum posterior portion
cc temp	corpus callosum temporal portion
cerped *	cerebral peduncle
chip *	cingulum bundle hippocampal portion
cing *	cingulum bundle dorsal portion
fimbria *	fimbria
fornix *	fornix
hipcom *	hippocampal commissure
ifof *	inferior occipital fasciculus
ilf *	inferior longitudinal fasciculus
intcap *	internal capsule
opticrad *	optic radiation
slfa *	superior longitudinal fasciculus portion A
slfb *	superior longitudinal fasciculus portion B
slfc *	superior longitudinal fasciculus portion C
slfd *	superior longitudinal fasciculus portion D
thalslat *	thalamic lateral projections
thalsmed *	thalamic medial projections
thalsub *	thalamic subcortical projections
vofa *	vertical occipital fasciculus portion A
vofb *	vertical occipital fasciculus portion B

* Bilateral tracts.

### 2.6 Harmonization and standardization

#### 2.6.1 Procedural harmonization

The EpiBioS4Rx preclinical rodent TBI model implemented comprehensive procedural harmonization across multiple field strengths and vendors, adjusting site-specific parameters to ensure uniform data. A pilot stage was conducted to minimize site-specific variations and ensure comparable image quality between the centers. Sequences were optimized on the 7T Bruker system at UEF and tailored for the different field strengths and gradient performances of the MRI systems at the other sites. Parameters such as TR, flip angle, and signal averages were adjusted to ensure comparable SNR between the sites (Immonen, Smith, et al., 2019).

#### 2.6.2 Diffusion signal harmonization

After preprocessing, a global statistics approach was utilized to harmonize the data across the three sites using the diffusion parameters signal intensities for all DTI parameters. Histograms of signal intensities for FA, RD, MD, and AD were generated to identify the peak value (mode), which typically reflects normal-appearing tissue. A global scaling factor was calculated as the reciprocal of the mode and applied to adjust parameter values to a common reference. This process includes averaging histograms for each rodent at each site to compute a harmonization factor, ensuring consistency and comparability of data across sites (Bhagavatula et al., 2023). Detailed procedure is described in Supplementary 2 (Supplementary Fig. 2).

#### 2.6.3 Standardization

After tractography, the DTI scalar values were standardized using z-scoring. This process involved calculating the mean (μ) and standard deviation (σ) for each DTI parameter (AD, MD, RD, FA) for the sham rat cases, and measuring the absolute z-score for each TBI case.

### 2.7 Data preparation

Out of the 47 fiber bundles, 11 fiber bundles had more than 10% missing values due to technical errors in streamline tractography. The rest of the fiber bundles containing missing values were imputed using k-nearest neighbors (k-NN) imputation (k = 5). For the subsequent analysis, 36 fiber bundles were used for each DTI metric.

### 2.8 Elastic net regularized regression

An ENR model was used to distinguish sham and TBI rats across four time points post-injury. The elastic net combines the Least Absolute Shrinkage Selector Operator (LASSO) penalty (Tibshirani, 1996) and the ridge regularization penalty (Hoerl & Kennard, 1970). The LASSO penalty reduces the coefficient estimate of unimportant predictors toward 0, making it suitable for our data with high dimensionality and relatively small size. In contrast, the ridge penalty is sensitive to multicollinearity (Zou & Hastie, 2005). The elastic net logistic regression equation is given by

$$\underset{\beta }{\min}\left(\frac{1}{2N}\sum_{i=1}^{N}\;\;{\left(y_{i}-\beta_{0}-\sum_{j=1}^{p}x_{ij}\beta_{j}\right)}^{2}+\lambda \left(\alpha |\beta {|}_{1}+\frac{1-\alpha }{2}|\beta {|}_{2}^{2}\right)\right),$$

where λ is the regularization parameter, α is the mixing parameter that controls the balance between L1 (Lasso) and L2 (Ridge) regularization. The goal of elastic net regression is to find coefficients that minimize the combination of the residual sum of squares (RSS) and the weighted sum of the L1 and L2 penalties.

We classified TBI versus sham rats using all the harmonized, z-scored, and k-NN imputed DTI metrics (FA, MD, RD, AD) of 36 fiber bundles as predictors. Model fitting was performed with a nested leave-one-out cross-validation (LOOCV) approach, a common evaluation strategy with limited datasets such as ours (Hastie et al., 2009). We used nested cross-validation, with the outer loop assessing the model performance and the inner loop performing hyperparameter optimization. Due to the imbalance between sham and TBI rats, class weights were adjusted to be inversely proportional to the outcome frequencies. Specifically, higher weights were assigned to the minority class (sham rats).

The model was iteratively trained on all data points for the outer loop, excluding one that served as the test case. The ENR model was fitted in an inner loop on the training set using the*cv.glmnet*function from the*glmnet*package (Tay et al., 2023) with an alpha value of 0.5. The function internally tunes the lambda (regularization parameter) value to minimize the mean cross-validated error. This error measure is computed based on the specified loss function (binomial deviance) to select the optimal lambda value.

The lambda (λ) value yielding the smallest inner cross-validated error was subsequently selected for prediction (TBI vs. sham rats) of the hold-out observation. The performance of the classifier was evaluated based on the area under the receiver operating characteristic curve (AUC), accuracy, sensitivity (the number of correctly classified TBI rats divided by the total number of rats), and specificity (the number of correctly classified sham rats divided by the total number of rats).

The predicted values, outcomes, and associated optimal lambda values are stored for each iteration of the LOOCV loop. After performing LOOCV, an ENR model was fitted using the entirety of the dataset. This was only done to extract optimal coefficients. The median lambda value across LOOCV folds was used. Predictor variables with nonzero coefficients were identified. The feature importance of the ENR models was defined as the absolute value of the regression coefficients. As the features are standardized, larger coefficient values correspond to higher importance (Zou & Hastie, 2005).

For each post-TBI time point (2 days, 9 days, 1 month, 5 months), we conducted a separate assessment. Analysis for each individual time point consisted of a different number of rats as given previously inTable 1. Within each fiber bundle, we evaluated all DTI metrics (mean FA, MD, RD, and MD) as individual predictor variables. This resulted in a total of 16 individual analyses, with each DTI metric being analyzed at each of the four time points.

Confidence intervals for the AUC were estimated using a non-parametric bootstrap approach with 1,000 bootstrap samples. For each bootstrap replicate, a sample with replacement was drawn from the original data, maintaining the sample size. The AUC was calculated for each replicate, and the 2.5th and 97.5th percentiles of the bootstrapped AUC distribution provided the bounds for a 95% confidence interval. This process was repeated for each DTI metric and time point, ensuring a robust estimation of the AUC confidence intervals (Zhu et al., 2010).

The model was implemented in R using the*glmnet*package (Friedman et al., 2010).

### 2.9 Statistical analysis

All statistical analyses were conducted in R (R version 4.1.3). Cohen’s d effect sizes were calculated for important fiber tracts between TBI and sham rats. In the figures, positive effect size values correspond to TBI having lower values than sham rats, whereas negative effect size values correspond to TBI having higher values relative to sham rats. The Kruskal–Wallis test followed by a post hoc analysis with Bonferroni correction to assess the differences between the sites in apnea duration. A three-way mixed-effects model with between-subject factors of site and outcome (TBI vs. sham rats), and time as a within-subject factor, was used to assess unharmonized and harmonized data. Similarly, a three-way mixed-effects model with between-subject factors of site and apnea, and time as a within-subject factor, was used to assess the effect of inter-site differences in apnea on DTI metrics in the TBI rats. A Spearman correlation test with false discovery rate (FDR) correction was used to assess the relationship between apnea duration and DTI metrics. The TBI rats were stratified into “High-TBI” and “Low-TBI” groups based on the median apnea score. A one-way ANOVA was conducted to compare the DTI metrics between the High-TBI, Low-TBI, and sham rats, followed by a Tukey HSD post hoc test.

## 3 Results

### 3.1 Bundle-specific tractography

A prototypical example of each bundle was extracted from the average multi-tensor sham rat data using manually guided seeding, inclusion, and exclusion. A few of the prototypical examples of tracts extracted from the average multi-tensor sham rat data are shown inFigure 2. Streamline tractography was not successful throughout due to possible technical errors, and fiber bundles which had more than 10% incomplete values (in total across all time points) were excluded from the analysis (Supplementary Table 3).

**Fig. 2.**
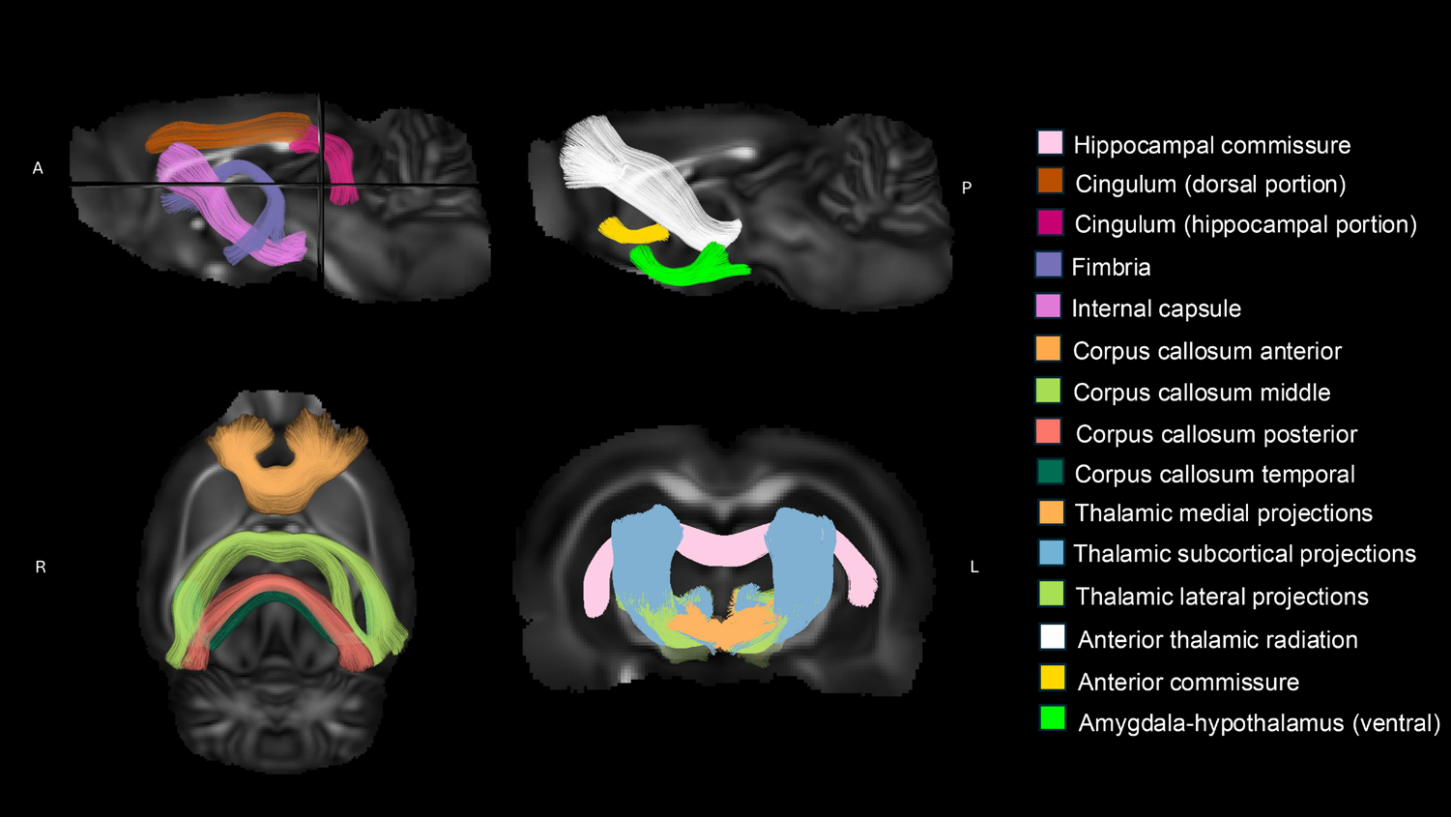
Prototypical example of tracts extracted from the average multi-tensor sham rat data used to generate tract orientation maps (TOM). A = anterior; L = left; P = posterior; R = right.

### 3.2 Classification performance

An elastic net regression model was used to classify sham and TBI rats based on harmonized and standardized DTI metrics (FA, RD, MD, AD) from 36 fiber bundles at 4 post-injury time points (2 days, 9 days, 1 month, 5 months). Model fitting was performed using a nested leave-one-out cross-validation (LOOCV) approach. The highest performance for each DTI metric was achieved with AD at 5 months (AUC = 0.972, 95% CI [0.945, 0.994]), RD at 5 months (AUC = 0.955, 95% CI [0.914, 0.984]), FA at 9 days (AUC = 0.915, 95% CI [0.862, 0.962]), and MD at 5 months (AUC = 0.941, 95% CI [0.897, 0.977]). When comparing across time points, the best performance at 2 days was with FA (AUC = 0.910, 95% CI [0.83, 0.96]), at 9 days with FA (AUC = 0.915, 95% CI [0.862, 0.962]), at 1 month with RD (AUC = 0.941, 95% CI [0.897, 0.977]), and at 5 months with AD (AUC = 0.972, 95% CI [0.945, 0.994]). AUC values remained above 0.86 at all other time points. The ROC curves and the cross-validated AUC values along with other performance metrics (accuracy, sensitivity, specificity, and confidence intervals) are shown inFigure 3.

**Fig. 3.**
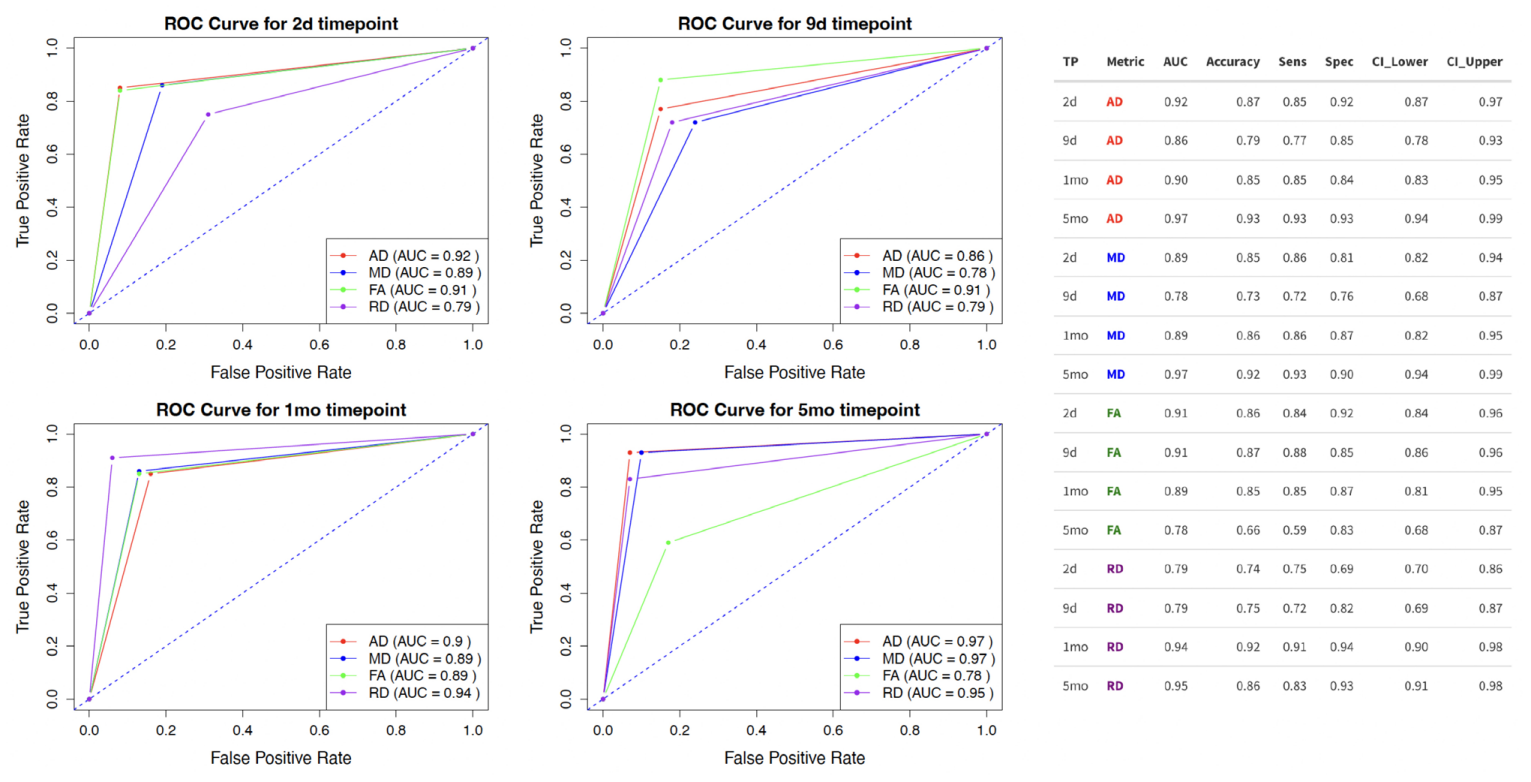
Receiver operating curves (ROCs) showcasing the classification performance of DTI metrics (AD, MD, FA, and RD) derived from 36 fiber bundles in differentiating TBI from sham rats at various post-injury time points (2 days, 9 days, 1 month, 5 months). The table to the right presents AUC values for each DTI measure at each time point, as well as accuracy, sensitivity (Sens), specificity (Spec), and lower and upper confidence intervals (CI).

### 3.3 Most discriminative fiber bundles

**Fig. 4.**
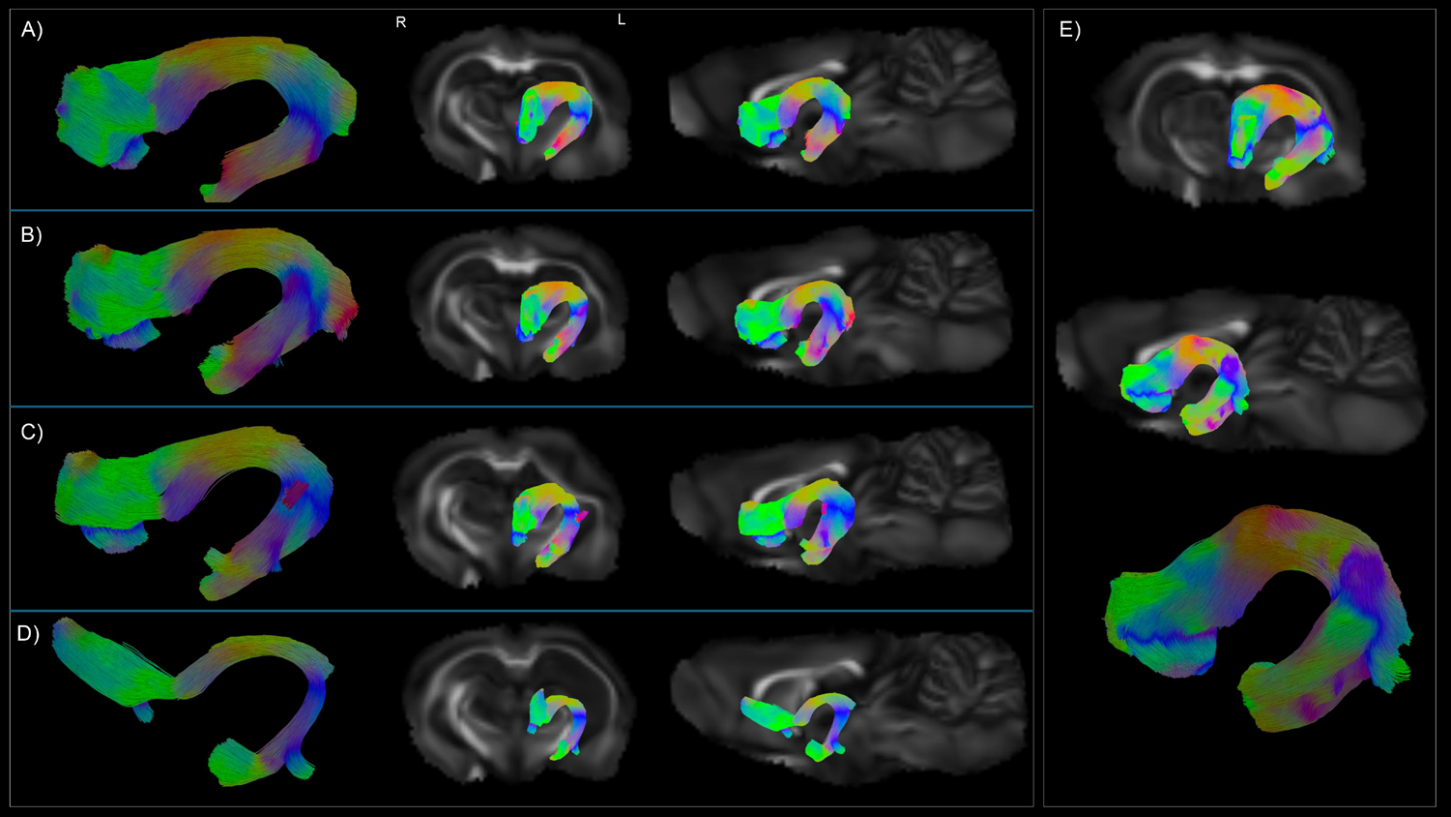
Tractography of the left fimbria in an example TBI rat at various time points post-injury: (A) 2 days, (B) 9 days, (C) 1 month, and (D) 5 months. These images illustrate the progressive microstructural alterations of the left fimbria following TBI. Panel (E) shows the tractography of the left fimbria in an example sham rat at 2 days post-injury, serving as a baseline for comparison. The colors represent the direction of water diffusion: red indicates left-right (medial-lateral) direction, green indicates anterior-posterior direction, and blue indicates superior-inferior (up-down) direction. L = left; R = right.

We assessed the total number of non-zero coefficients selected by the ENR model, which is indicative of their classification capacity across all DTI metrics. All predictors were ipsilateral to the injury site, with the left fimbria, left thalamic subcortical projection, and the left internal capsule exhibiting the highest discriminative power, consistently emerging as top predictors.Figure 4shows disruptions in the left fimbria tractography in native space over 4 time points post-TBI: 2 days, 9 days, 1 month, and 5 months, for an example TBI rat along with a comparison with an example sham rat at 2 days post-injury. The absolute coefficient value of the top predictors for each DTI metric for each time point is shown inFigure 5, alongside prototypical examples of the left fimbria, thalamic subcortical projection, and internal capsule.

**Fig. 5.**
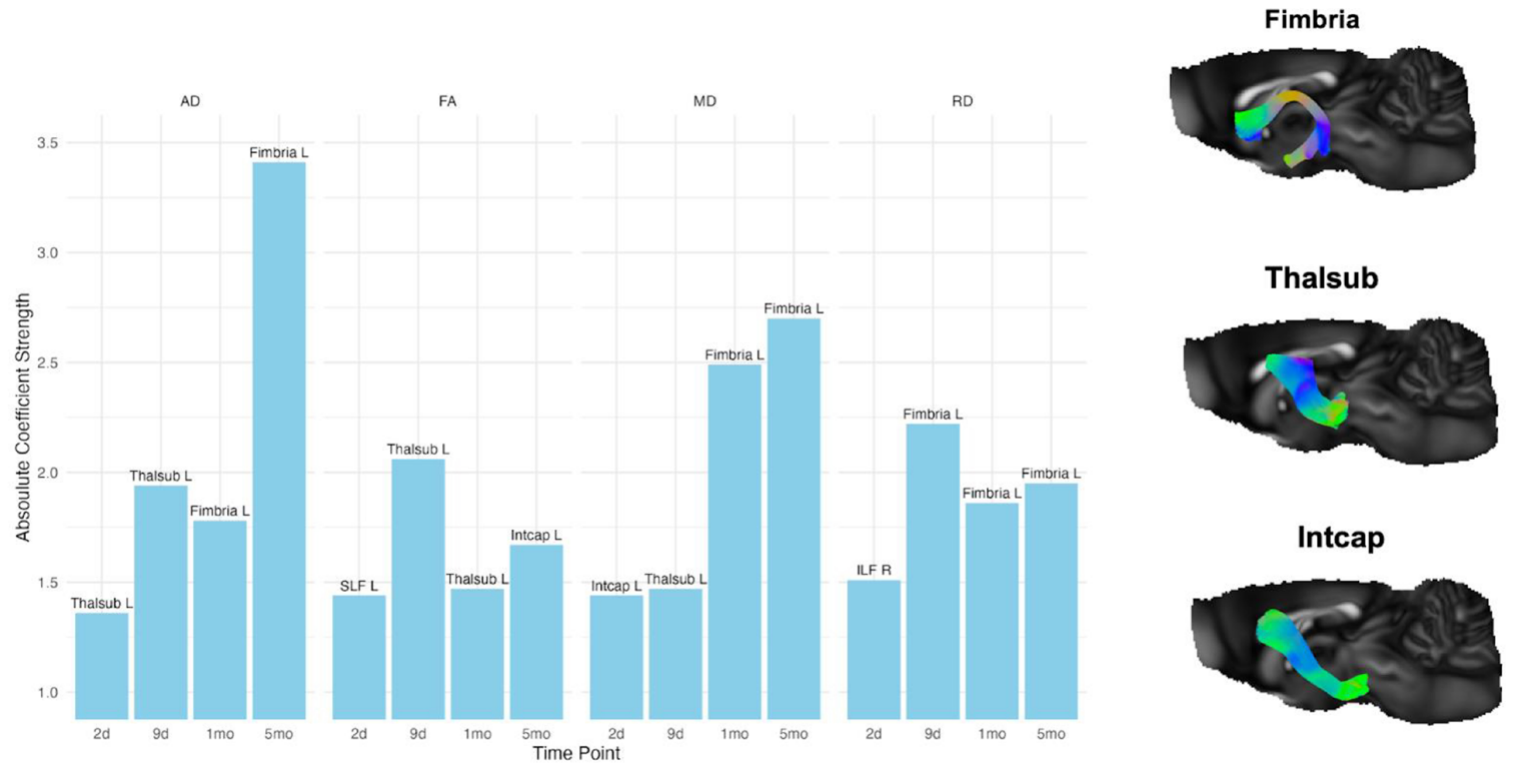
Top predictors for differentiating TBI from sham rats for each time point and DTI metric as determined by the absolute coefficient value. ILF = inferior longitudinal fasciculus; Intcap = internal capsule; L = left; R = right; SLF = superior longitudinal fasciculus portion; Thalsub = thalamic subcortical projections. Prototypical example of the left fimbria, thalamic projection, and internal capsule extracted from the average multi-tensor sham rat data.

### 3.4 Effect sizes

**Fig. 6.**
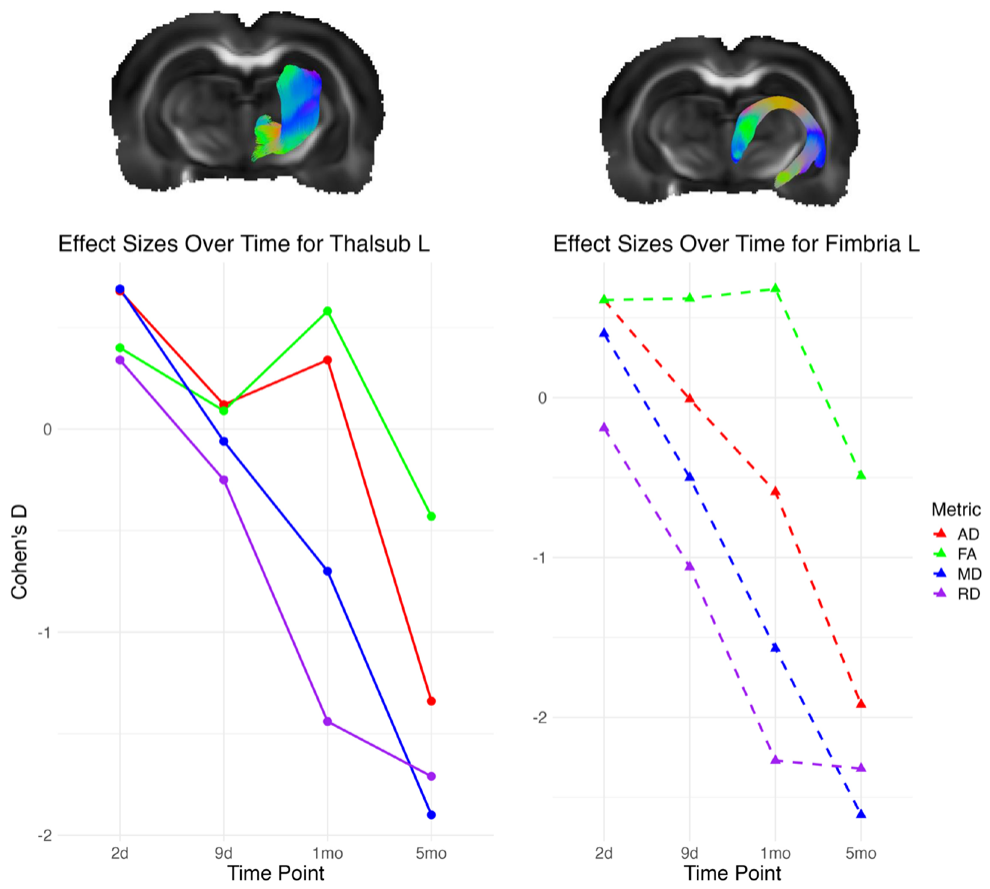
Effect size line charts comparing sham versus TBI rats for each DTI metric at each time point for the left thalamic subcortical projection (Thalsub L; solid line) and left fimbria (Fimbria L; dashed line).

To provide a more interpretable measure of the strength and translational significance, we calculated the effect sizes of top predictors: left fimbria and left thalamic subcortical projections at each time point. The largest effect size corresponded to MD values at 5 months in the left fimbria (Cohen’s d = -2.61), and the left thalamic subcortical projections showed a large effect size (Cohen’s d = -1.9). These negative values of Cohen’s d indicate that values were higher in TBI rats than in the sham rats. Generally, on the early post-TBI phases (2 and 9 days), effect sizes were small to moderate compared with the later time points (1 and 5 months) (Fig. 6). All effect sizes are reported inSupplementary Table 4.

### 3.5 Harmonization

**Fig. 7.**
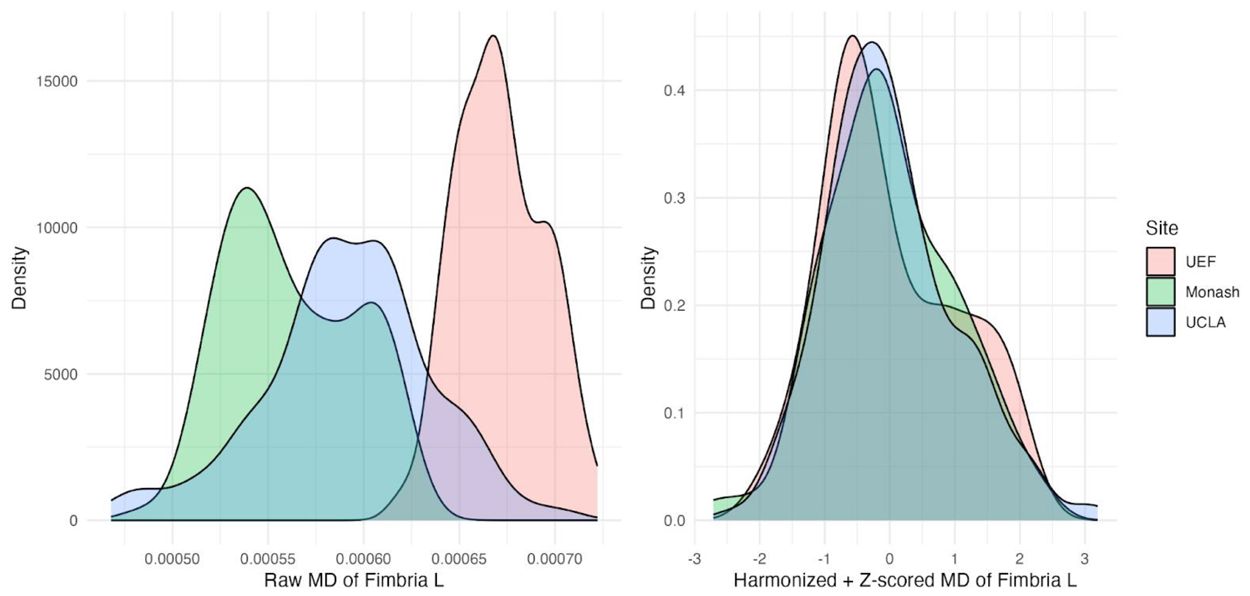
Comparison of pre- and post-harmonization and z-scored density plots of left fimbria MD values across sites. Density plots of the mean MD values of the left fimbria at 2 days time point from each site (left) before and (right) including both TBI and sham rats after harmonization and z-score standardization and harmonization.

To assess the effect of harmonization, we analyzed the left fimbria MD values at 2 days as this DTI metric and region displayed the highest sensitivity to TBI pathology. Mixed-effect analysis of the raw left fimbria MD values showed a significant effect of site (estimate = -1.021e-04, Std. Error = 1.449e-05, t value = -7.050, p-value < 0.001) and outcome (Estimate = 2.995e-05, Std. Error = 1.021e-05, t value = 2.934, p-value = 0.004). Post-standardization and harmonization removed the effect of site, indicating successful mitigation of site-specific variability. Post-harmonization density plots of left fimbria MD values (Fig. 7, right), in contrast to pre-harmonization (Fig. 7, left), demonstrate a notable convergence of MD value distributions across sites.

### 3.6 Effect of apnea duration and site

**Fig. 8.**
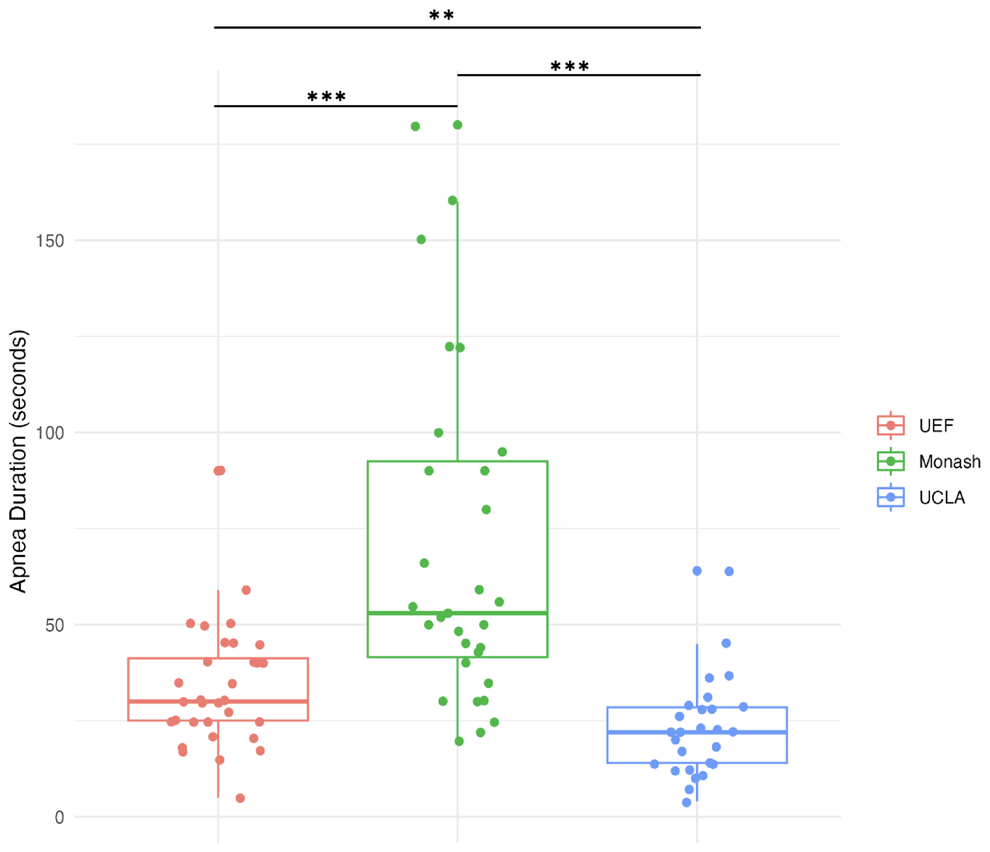
Inter-site variability in apnea duration among TBI rats. The box plot illustrates the distribution of apnea duration across different research sites (UEF, UCLA, and Monash) within the subset of TBI rats. Significant inter-site variability is highlighted.

Previous data presented byNdode-Ekane et al. (2024), assessing inter-site variability in apnea duration among TBI rats in the EpiBioS4Rx cohort, revealed significant differences. Consistent with these prior findings, the Kruskal–Wallis test revealed significant inter-site differences in apnea duration (p < 0.001). A post hoc analysis revealed UEF rats had significantly lower apnea durations than Monash (p < 0.001). In UCLA rats, the apnea duration was shorter than in Monash (p < 0.001) and UEF (p < 0.05) (Fig. 8).

The goal of our exploratory analysis was to understand how inter-site variability in apnea duration may influence microstructural integrity and diffusivity, potentially contributing to variations in WM characteristics observed in TBI outcomes. A three-way mixed-effects analysis for the FA and MD values of the top predictors (left fimbria and the left thalamic subcortical projections showed that there was neither a significant main effect of apnea nor site, nor were there significant interactions between site and apnea across all time points.

### 3.7 Exploratory analysis

**Fig. 9.**
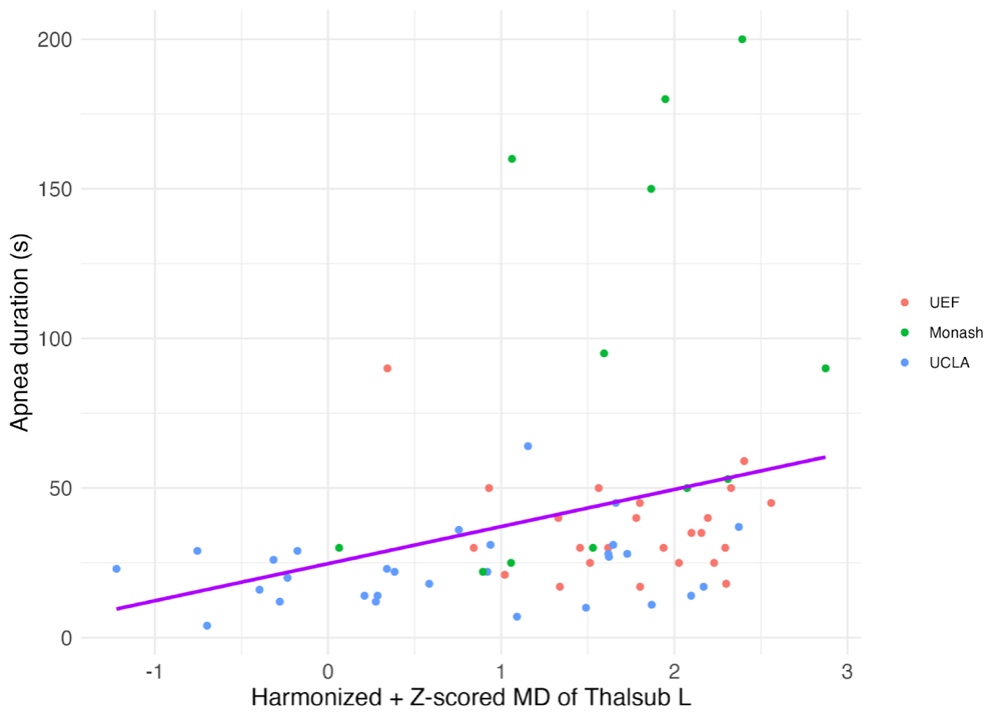
Significant correlation between apnea duration and the harmonized z-scored MD values in left thalamic subcortical projections of TBI rats at 5 months post-injury. Correlation analysis, adjusted for multiple comparisons using FDR correction, revealed a significant relationship (r = 0.44, p-adjusted < 0.001), indicating that higher apnea duration is associated with increased MD values in TBI rats.

A correlation analysis was performed between each DTI metric’s top predictor (left fimbria and left thalamic subcortical projections) at each time point and the apnea duration, correcting for multiple comparisons with FDR correction. The only significant correlation with apnea duration was found with harmonized z-scored MD values of the left thalamic subcortical projections at 5 months post-injury (r = 0.43, p-adjusted < 0.001) (Fig. 9), indicating higher apnea duration is correlated with higher MD values of the left thalamic subcortical projections in the TBI rats 5 months post-injury. We removed the upper-bound outliers for apnea duration and the correlation remained significant (r = 0.41, p < 0.001). Other significant correlations prior to FDR correction include a positive correlation with AD values of the left thalamic subcortical projections at 5 months, AD values of the left fimbria at 1 month, MD values of the left fimbria at 1 month, RD values of the left fimbria at 2 days and 1 month. All correlations are shown inSupplementary Table 5.

To further examine the influence of apnea on DTI metrics, the TBI rats were stratified into two groups based on apnea duration recorded post-injury: “TBI-Low” and “TBI-High,” using the median apnea duration (30 s). A one-way ANOVA was conducted to compare the harmonized z-scored MD values in the left thalamic subcortical projections between the TBI-Low, TBI-High rats, and sham rats at 5 months post-injury. The analysis revealed a significant difference in MD values in the left thalamic subcortical projections (p < 0.001) (Fig. 9, right). A post hoc analysis using Tukey’s Honest Significant Difference (HSD) test showed that the Low-TBI group had significantly lower MD values than the High-TBI group (adjusted p-value < 0.001). Furthermore, the sham group exhibited significantly lower MD values than both the High-TBI (adjusted p-value < 0.00001) and Low-TBI groups (adjusted p-value < 0.001). The results suggest that the duration of apnea is associated with greater alterations in MD values of the left thalamic projections at 5 months post-injury.

Further we visualized the left thalamic subcortical projection of an example rat from each of the stratified groups at 5 months post-injury: (i) High-TBI: site = Monash; id = 2401; apnea = 200 s, (ii) Low-TBI: site = UCLA; id = 3152; apnea = 22 s, and (iii) sham: site = UEF; id = 1146; apnea = NA. The TBI rat with high apnea scores (Fig. 9, left, top) appears to have more left-right oriented white matter fibers, suggested by the increased presence of red color in the tractography (Pajevic et al., 1999). In contrast, the sham rat (Fig. 9, left, bottom) appears to have a more uniform and organized structure and the color-coding indicating fiber orientations is more consistent and evenly distributed.

## 4 Discussion

This study, as part of the multisite pre-clinical EpiBioS4Rx, evaluated the utility of a computational framework which includes components of artifact correction, multi-compartmental modeling, tensor-based spatial normalization, tractography, and harmonization in detecting TBI-induced white matter microstructural changes over time. The ENR regularization model effectively distinguished TBI rats from sham rats by detecting microstructural changes in white matter at 2 days, 9 days, 1 month, and 5 months post-injury. To the best of our knowledge, this study represents the first application of ENR in conjunction with bundle-specific tractography to detect WM microstructural abnormalities in a longitudinal multi-site rat model of TBI. Our findings serve as proof-of-concept for methodologies that will be integrated into EpiBioS4Rx to distinguish between TBI and PTE rats in future research.

We used a novel kernel regression estimation of fiber orientation mixtures and bundle-specific tractography approach that combines statistically robust tractography analysis with advanced methods for selecting and analyzing major fiber bundles. This analysis allows for quantitative characterization of entire bundles and provides an informative assessment of TBI-induced pathologies. Standard DTI is limited in resolving crossing fibers and is prone to partial volume effects (Assaf & Pasternak, 2008). Our method can differentiate between multiple fiber populations, providing a more accurate representation of underlying anatomy. Specifically, within the context of EpiBioS4Rx, the high sensitivity and specificity of bundle-specific modeling make it well suited for analyzing the injury process, allowing for the identification of injury biomarkers, or evaluating the effectiveness of therapeutic interventions.

Tractography in the context of TBI is prone to technical limitations due to the added complexity of injury-induced pathology (Harris et al., 2016;Yeh et al., 2021). Our method was unable to analyze certain tracts in some of the rats. The percentage of failures in these tracts was consistently higher in TBI rats than in sham rats: thalslat right (sham = 2%, TBI = 13.3%), thalslat left (sham = 6%, TBI = 20%), cc temp (sham = 24.4%, TBI = 71.1%), thalsmed left (sham = 8.8%, TBI = 35.5%), thalsmed right (sham = 11.1%, TBI = 44.4%) (Supplementary Table 1). Our analysis includes significant underrepresentation of lateral and medial thalamic and CC damage in the TBI group. We acknowledge that this causes selective omission and introduces a biased estimation of the overall TBI-induced WM trajectory. Our future methods will attempt to integrate histology and multimodal MRI to combat the complexities introduced by TBI.

The most substantial structural and microstructural alterations were observed in the ipsilateral region, consistent with lateral LFPI results reflecting the more prominent pathophysiological impact at the site of injury (Immonen, Harris, et al., 2019). The model was particularly sensitive to changes in the fimbria and thalamic projections ipsilateral to the injury site, which are bundles commonly affected in rat lateral FPI models (Shultz et al., 2015;Willats et al., 2014;Wright et al., 2016). Of the DTI metrics, AD at 5 months post-injury demonstrated the highest predictive capacity (AUC = 0.97), underscoring the late-stage microstructural changes associated with TBI. Similarly, MD and RD demonstrated heightened sensitivity to TBI pathology at 1 and 5 months post-injury. However, FA features demonstrated its highest predictive capacity at early stages (2 and 9 days). The impact of LFPI results in pathophysiological consequences that cause substantial neuronal loss, vascular damage, axonal injury, neuroinflammation, and protein aggregation, many of which develop over a prolonged period (Bao et al., 2012;Flygt et al., 2013;Kharatishvili & Pitkanen, 2010;Shultz et al., 2013,^2015^;Wright et al., 2017). We observed a pattern of WM alteration that aligns with the general pathophysiology of TBI (Harris et al., 2016;Webster et al., 2015;Wright et al., 2017).

Post-classification, our secondary analysis focusing on the left fimbria and thalamic subcortical projection suggests increased tissue damage and demyelination at 5 months as reflected by increased MD and RD (Harris et al., 2022;Mazrooyisebdani et al., 2020). Alongside, the increased AD may be representative of axonal repair and remodeling. The tractography results illustrate a trajectory of degeneration in the left fimbria following TBI, evidenced by the increasing fragmentation and decreasing density of the tracts, which may corroborate with ongoing axonal repair and remodeling. However, it is important to note that anisotropy is influenced differently depending on the changes in myelination and myelinated fibers in a region-specific manner (Fathy et al., 2019;Harris et al., 2016;Hutchinson et al., 2017;Laitinen et al., 2015). TBI causes complex and widespread axonal pathology and structural reorganization which requires careful interpretation. Our follow-up study will utilize other imaging modalities and techniques to supplement the understanding of the underlying pathological process captured by our method.

Our results showed that the ipsilateral thalamic subcortical fiber bundle is particularly vulnerable to the effect of TBI severity. Several studies have highlighted the significance of thalamic damage in TBI (Little et al., 2010;Scott et al., 2015). The thalamus is a major relay center of cortical fibers, and damage to these projections may result in widespread functional impairments (Scott et al., 2015). Discerning the delayed manifestation of this effect offers a window for therapeutic interventions and emphasizes the need for long-term monitoring of individuals with TBI. A previous single-site rat study in UEF demonstrated the potential of acute thalamic damage as a prognostic marker for PTE (Manninen et al., 2021). Our future research will attempt to understand the relationship between TBI severity, thalamic projection damage, and the risk of developing PTE.

**Fig. 10.**
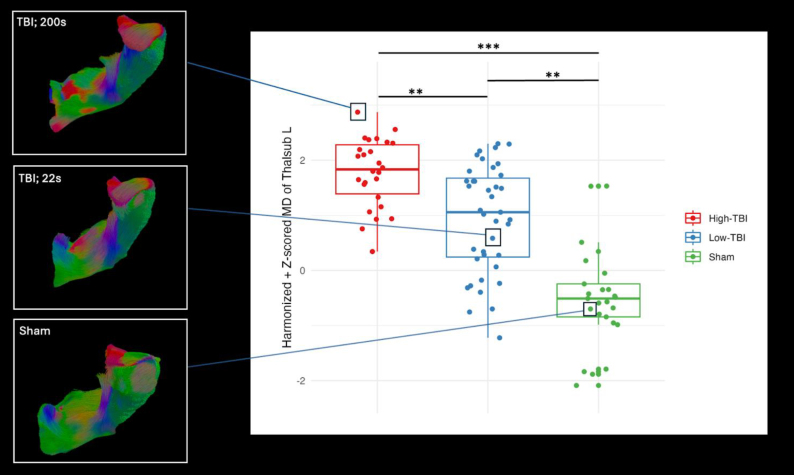
[Right] Comparison of harmonized z-scored mean diffusivity (MD) of left thalamic subcortical projections among TBI rats stratified by apnea duration and sham rats at 5 months post-injury. TBI rats were divided into “TBI-Low” and “TBI-High” groups based on median apnea duration. A one-way ANOVA highlighted significant differences in MD values due to stratified injury severity (p < 0.001), with post hoc Tukey’s HSD test revealing lower MD values in “TBI-Low” compared with “TBI-High” (adjusted p < 0.001), and both TBI groups showing increased MD values compared with sham rats (High-TBI vs. sham adjusted p < 0.00001; Low-TBI vs. sham adjusted p < 0.001). [Left] From bottom to top, tractography reconstruction of the left thalamic subcortical projections demonstrating the structural changes in an example “High-TBI” rat with 200 s apnea duration, an example “Low-TBI” rat with 22 s apnea duration, and an example sham rat. The colors represent the direction of water diffusion: red indicates left-right (medial-lateral) direction, green indicates anterior-posterior direction, and blue indicates superior-inferior (up-down) direction. Asterisks indicate statistical significance: *p< 0.05, **p< 0.01, ***p< 0.001, ****p< 0.0001.

Our exploratory analysis showed that increased apnea duration is associated with increased MD values of the left thalamic subcortical projections at 5 months. The duration of the transient apnea has been suggested as an indicator of injury severity (Igarashi et al., 2007;Ndode-Ekane et al., 2019). Our stratification analysis showed that TBI rats with higher apnea duration had a more prominent effect on MD values than the TBI rats with lower apnea duration and sham rats at 5 months (Fig. 10, right). The 5 months period post-injury showed the most robust differences in MD values between the sham and TBI rats, with TBI rats having increased MD values, which our model was sensitive to. Tractography visualization revealed that rats with longer apnea times showed greater WM fiber alterations compared to both TBI rats with shorter apnea duration and sham rats, which could explain the differences between groups (Fig. 10). Higher MD values suggest increased tissue damage, edema, or increased extracellular space, which may lead to inconsistent and nonuniform fiber organization, indicating more severe white matter disruption (Shenton et al., 2012;Xiong et al., 2014). The results validate the biological significance of apnea duration as a marker for TBI severity and suggest that it has a region-specific effect that manifests at later time points post-injury.

Comparing our results with previous similar rat TBI models, we found classification performance could vary depending on the choice of machine learning algorithms and neuroimaging feature utilities (Bostami et al., 2022;Vishwanath et al., 2020). A prior investigation using random forests and hippocampal position and orientation features, focusing exclusively on the UEF EpiBioS4Rx sub-cohort, reported balanced accuracies of 98%, 62%, 93%, and 89%, paired with AUC values of 0.98, 0.69, 0.98, and 0.96 at 2 days, 9 days, 1 month, and 5 months post-TBI, respectively (De Feo et al., 2022). The same study evaluated rats from another cohort and reported balanced accuracies of 91%, 92%, and 98%, complemented by AUC values of 0.97, 0.96, and 0.99 at 2, 7, and 21 days post-TBI, respectively. In comparison with human TBI studies,Vergara et al. (2017)used a linear support vector machine and classified mild TBI (mTBI) in humans with an accuracy of 84% using resting-state functional network connectivity features, 75% using dMRI features, and 74.5% using both. In contrast,Mitra et al. (2016)utilized a random forest classifier and achieved a classification accuracy of 67.16% using FA features from structural connections from network-based statistics. Another study classified TBI in humans with an accuracy of 66%, utilizing the standard DTI metrics of WM structures and random forests (Sharma et al., 2021). By comparing and considering disparities in methodology, feature sets, and cohort diversity, our multi-site analysis achieved consistently high performance, underscoring its robustness in the context of increased data variability and the potential scalability and reliability of this method (Jiang et al., 2022;Parsaei et al., 2023).

There was no effect of site on the DTI metrics post-standardization and harmonization, indicating the effectiveness of the harmonization process, consistent with our previous work evaluating the effectiveness of the harmonization method (Bhagavatula et al., 2023). Taken together, our analyses provide evidence that despite the observed inter-site variability in apnea, the effect of apnea is consistent across sites: apnea’s impact on DTI metrics is relatively independent of the inter-site differences in apnea duration. This is further supported by the observed significant correlation between apnea duration and MD values after accounting for outliers. Previous EpiBioS4Rx studies on procedural harmonization have shown that variability in the injury parameters may be attributed less to site-specific experimental protocol differences, and to a greater extent to site-specific environmental influences such as individual differences in response to injury, genetic backgrounds, and environmental factors unique to each site (Ndode-Ekane et al., 2019,^2024^). This suggests that our harmonization framework is effective in maintaining biological variability associated with TBI. All the study sites agreed on common data elements (CDEs) to ensure a standardized system of data collection for every rat in all sites (Pitkänen et al., 2019). Our results highlight the rigorous efforts and success of the EpiBioS4Rx participating centers in Finland (UEF), Australia (Monash), and USA (UCLA) in harmonizing the methodological protocols, and data collection using CDEs.

Preserving the inherent biological variability post-harmonization is essential as it can be leveraged to model the genetic and exposomal heterogeneity observed in human TBI (Duncan et al., 2018;Garner et al., 2019;Shultz et al., 2017), thereby addressing the challenge of heterogeneity of preclinical and clinical TBI and PTE (DeWitt et al., 2018). For a potential MRI biomarker for PTE to be viable for clinical translation, it must capture robust TBI-induced structural changes resilient to the inherent methodological variability across sites (Immonen, Harris, et al., 2019). However, it is important to consider that harmonization could mask or reduce biological variance (Fortin et al., 2017). While harmonization is crucial for ensuring data comparison across sites, researchers must also consider the potential for preserving biological variability and the need for analytical approaches that can detect subtle, site-specific effects (Orlhac et al., 2022;Pinto et al., 2020). Our recent work demonstrates that ComBat harmonization, an empirical Bayes approach, can harmonize multi-site data while preserving injury effects (Quach et al., 2024). In the broader context of multi-site studies, the EpiBioS4Rx studies highlight the challenges and considerations in conducting and analyzing multi-site neuroimaging studies (Lutkenhoff et al., 2020;Ndode-Ekane et al., 2019,^2024^;Quach et al., 2024).

While our findings demonstrate the utility of various DTI metrics in assessing TBI-induced changes, it is important to acknowledge potential limitations in their interpretation, particularly regarding RD. Other neuroinflammatory processes, such as microglial activation and cerebral edema, can alter diffusion dynamics and contribute to RD increases (Budde et al., 2011;Pasternak et al., 2015), making it challenging to distinguish between actual tissue damage and transient injury response processes. Additionally, structural reorganization after TBI can influence RD due to remodeling of axonal pathways and compensatory mechanisms such as axonal sprouting (Harris et al., 2016). To fully understand the dynamic changes in RD, it is crucial to integrate other DTI metrics such as FA, AD, and MD. These metrics provide complementary information that helps to interpret RD changes more accurately during both the acute injury response and long-term recovery. For example, while increased RD may signal demyelination, decreased FA, AD, and increases in MD can be attributed to microstructural changes, such as the loss of myelinated axons and/or the accumulation of iron deposits (Wang et al., 2024). By examining these metrics together, we can achieve a more comprehensive assessment of white matter microstructural integrity and ensure that RD changes are contextualized within broader patterns of structural reorganization, aiding in the interpretation of both acute responses and long-term recovery processes.

Our study has a few other limitations. Although our study utilized multiple DTI metrics, other dMRI models or combinations such as apparent fiber density (AFD) and track-weighted imaging (TWI) metrics could provide further insights into TBI pathology (Wright et al., 2017). Relying solely on the described metrics may exclude potentially valuable information. Additionally, the study observed rats at specific time points post-injury, and the results were inherently restricted to this defined time frame. Future studies should explore chronic alterations beyond this time frame. For our exploratory analysis, we only utilize one injury outcome: apnea time. However, there are other acute injury parameters such as righting reflex, neuroscores, lesion cavity that could provide additional information (Ndode-Ekane et al., 2019,^2024^). Only male rats were used in this study, as the study was not designed to analyze sex differences. However, studies have shown that female rats may exhibit distinct injury responses and recovery trajectories (Rubin et al., 2019). Future preclinical research incorporating both sexes are required to characterize the nature of sex-dependent injury and recovery. Finally, our dataset consisted of a class imbalance with more TBI rats than sham rats, which may be attributed to the variable sensitivity in the model across all DTI metrics (0.50–0.93). During regularization, even after assigning class weights, the model could over-penalize features that have predictive power for the minority class and can cause the model to be biased toward the majority class (TBI rats), leading to a higher number of false negatives. The model may prioritize classifying instances from the majority class at the expense of effectively identifying the minority class (Luque et al., 2019).

## 5 Conclusion

This study demonstrates the diagnostic potential of our framework utilizing bundle-specific tractography analysis in conjunction with elastic net regression to identify and delineate the dynamic behavior of DTI metrics over time, including white matter microstructural variances, enabling accurate differentiation between TBI and sham rats, and elucidating the periods during which each metric potentially offers the most diagnostic utility. Detecting the acute and chronic phases of injury has implications for early detection, monitoring, understanding the pathophysiology of the injury, and personalizing patient care. Our multi-site, harmonized framework enhances generalizability, addressing the intrinsic variability associated with TBI and aligning with the EpiBioS4Rx study’s objective of developing a reliable predictive model for identifying PTE biomarkers and evaluating therapeutic interventions. This approach is fundamental for designing targeted clinical trials and personalized treatment plans, thereby advancing the management and rehabilitation paradigms. Our future research will extend this framework to characterize and identify biomarkers of PTE (Mishra et al., 2011).

## Data and Code Availability

The code for bundle-specific tractography and elastic net prediction is available upon request.

## Author Contributions

Richard J. Staba, Terence J. O’Brien, Asla Pitkanen, and Dominique Duncan designed the study. Xavier Ekolle Ndode-Ekane, Pedro Andrade, Riikka Immonen, Eppu Manninen, Noora Puhakka, Mette Heiskanen, Neil G. Harris, Gregory D. Smith, Cesar E. Santana-Gomez, Richard J. Stab, David K. Wright, Idrish Ali, Nigel C. Jones, Sandy R. Shultz, Pablo M. Casillas-Espinosa, Matthew R. Hudson, Juliana Silva, Glenn Yamakawa, and Emma L. Braine set up the methodologies, performed conceptual and experimental design, acquisition, analyzed the procedures-related and physiological data, analyzed the EEG data, and contributed to interpretation of results and editing of the manuscript. Alexis Bennett, Sweta Bhagavatula, Rachael Garner, and Tuba Asifriyaz contributed to the preprocessing, quality control, and interpretation of results and editing of the manuscript. Ryan Cabeen developed the preprocessing pipeline, and bundle-specific tractography. Akul Sharma generated and analyzed the MRI data and wrote the manuscript.

## Declaration of Competing Interest

The authors declare that they have no competing interests.

## Ethics

UEF: All animal procedures were approved by the Animal Ethics Committee of the Provincial Government of The Southern Finland and carried out in accordance with the guidelines of the European Community Council Directives 2010/63/EU. Monash: All animal procedures were approved by the Florey Animal Ethics Committee (ethics number 17-014 UM) at the University of Melbourne and by the Alfred Medical Research & Education Precinct Animal Ethics Committee (E/1799/2018/M) at the Monash University. UCLA: All animal procedures were approved by the University of California Los Angeles Institutional Animal Care and Use Committee (protocol 2000-153-61 A).

## Supplementary Material

Supplementary Material
