# Supplementary material for: Bundle-specific tractography approach for identifying white matter microstructural changes following traumatic brain injury in rats: An EpiBioS4Rx study
Source: Imaging Neurosci (Camb). 2025 Feb 18;3:imag_a_00471. doi: 10.1162/imag_a_00471 (PMC12319758; doi:10.1162/imag_a_00471)
Supplement: Supplementary Material [file imag_a_00471-supp.pdf]

Supplement Table 1: List of procedures, equipment, and suppliers used for TBI induction at different sites: University of Eastern Finland (UEF), Monash University, and the University of California, Los Angeles (UCLA). Adapted from: Ndode-Ekane, X. E., Ali, I., Santana-Gomez, C. E., Casillas-Espinosa, P. M., Andrade, P., Smith, G., ... & Pitkänen, A. (2024). Successful harmonization in EpiBioS4Rx biomarker study on post-traumatic epilepsy paves the way towards powered preclinical multicenter studies. Epilepsy Research, 199, 107263.

Category	Site	Catalog Number	Vendor	Country
Rats	UEF	Sprague Dawley (SD)	Envigo Laboratories B.V.	The Netherlands
	Monash	Sprague Dawley (SD), In house bred	Animal Research Platform (MARP) Monash University	Australia
	UCLA	Sprague Dawley (SD)	Charles River	USA
Induction of TBI				
Anesthesia System	UEF/Monash	Somnosuite # SS6069B	Kent Scientific	USA
	UCLA	Matrix VIP 3000 Vaporizer # 91305430	Patterson Veterinary	USA
Trephine	UEF/UCLA	#18004–50	Fine Science Tools GmbH	Germany
	Monash	Dremmel Model 300	Dremmel Model 300	Australia
Tissue Adhesive	UEF/UCLA	3M Vetbond	3M Deutschland GmbH	Germany
	Monash	Octyl cyanoacrylate	Bostik	Australia
Dental Acrylate	UEF	Selectaplus #10009210, Selectaplus #D10009102	DeguDent	Germany
	Monash	AVSCV00500	Vertex	The Netherlands
	UCLA	SNAP Liquid (P16–02–65), SNAP Powder (P16–02–60)	Pearson Dental	USA
Fluid Percussion Device	All Sites	Model FP 302	AmScien Instruments	USA
Postoperative Care				

Analgesic	UEF	Buprenorphine	Orion Pharma	Finland
	Monash	Buprenorphine	Indivior Pty Ltd	Australia
	UCLA	Flunixin meglumine	MERK	USA
Additional Analgesic	UEF/Monash	Not applicable	Not applicable	Not applicable
	UCLA	Flu-Nix Flunixin Meglumine	AgriLabs	USA
Medical Oxygen	UEF/UCLA	Not applicable	Not applicable	Not applicable
	Monash	Not applicable	Mediquip Medical Equipment & Supplies	Australia
Antibiotics	UEF/Monash	Not applicable	Not applicable	Not applicable
	UCLA	Trimethoprim sulfamethoxazole (TMS) medicated rodent chow	Envigo Laboratories	USA
Food Pellet	UEF	2016S (Teklan Diet)	Envigo Laboratories B.V.	The Netherlands
	Monash	102108	Barastoc	Australia
	UCLA	LabDiet 5001	LabDiet	MO, USA
Food Supplement	UEF/UCLA	Not applicable	Not applicable	Not applicable
	Monash	Powder milk	Sustagen, Nestle	Australia

Supplement 2: Inter-Site Diffusion Data Harmonization

A global statistics approach was utilized to harmonize the data across 3 sites using the diffusion parameters signal intensities. After preprocessing and parameter estimation, a histogram is generated of the signal intensities of each parameter (FA, RD, MD, AD) and the peak value or mode is identified (Figure 1, left). The mode represents the most common parameter value, typically corresponding to normal-appearing tissue. A global scaling factor is calculated as the reciprocal of the mode (i.e., scaling factor = 1 / mode) and applied to the parameter values to bring the mode to a value of one and subsequent histograms are generated (Figure 1, right). Histograms for each rat within each site are averaged to compute the harmonization factor for site-specific adjustments and generate a site representative histogram. This ensures that the data from each site is scaled appropriately to bring the mode to a common reference value, making the data more consistent and comparable across different sites. The mode is

used instead of the median to overcome bias due to lesion size. Lesions occupy a smaller volume compared to normal brain tissue and will appear less on the histogram and do not influence the mode but may change the median depending on size and distribution. This method reduces variability due to individual differences and lesions, making the data more consistent and comparable across rats.

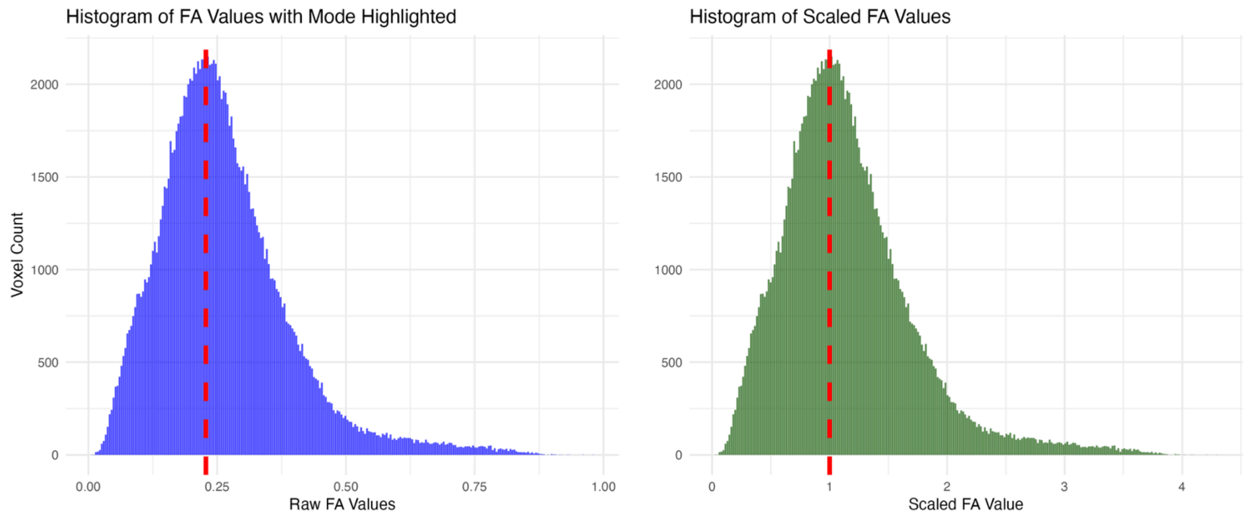

Supplement Figure 2. Left: Histogram of the FA values of the whole brain scan of an example rodent. Right: Histogram of the example rodent after applying a scaling factor of 4.38.

Supplement Table 3: Summary of the failure for streamline tractography at each timepoint. This table summarizes the percentage of missing values for various fiber bundles, outcome groups (Sham and TBI). The percentages indicate the proportion of missing data for each tract, providing insights into data completeness across different timepoints. The following tracts were excluded: temporal part of the corpus callosum (CC temp), lateral and medial portion of the left and right thalamic projections (thalslat and thalsmed), the left B-portion of the superior longitudinal fasciculus (slf B) and the right D-portion (slf D), left vertical occipital fasciculus (vofa and vofb), and the dorsal connection of the amygdala-hypothalamus (amyhypdor).

Tract	Sham (% NA)	TBI (% NA)
Amyhypdor L	67.18	84.97
Amyhypven L	0	0.82
Amyhypven R	0.76	0.27
Ant Comm	1.53	1.37

Atr L	1.53	0.82
Atr R	0.76	0.82
CC Ant	0.76	0.55
CC Mid	42.75	75.41
CC Post	4.58	7.1
CC Temp	80.15	79.23
Cerped L	1.53	1.09
Cerped R	1.53	1.09
Chip L	0.76	5.46
Chip R	3.05	3.01
Cingulum L	0.76	0.55
Cingulum R	0.76	1.91
Fimbria L	0.76	1.37
Fimbria R	0.76	0.82
Fornix L	0.76	1.64
Fornix R	1.53	0.82
Hipcom	3.05	13.39
Ifof L	2.29	1.09
Ifof R	7.63	4.64
Ilf L	0	0.55
Ilf R	0.76	0
Internal Capsule L	0.76	0.82
Internal Capsule R	0.76	0.82
Opticrad L	12.98	22.68
Opticrad R	8.4	6.56
Slfa L	1.53	0
Slfa R	0	0
Slfb L	0.76	0
Slfb R	0.76	0
Slfc L	19.08	24.59
Slfc R	8.4	4.64
Slfd L	31.3	55.19
Slfd R	14.5	11.2
Thalamus lateral L	17.56	16.67
Thalamus latera R	12.21	10.38
Thalamus medial L	25.19	30.05
Thalamus medial R	33.59	33.88

Thalamus subcortical L	0.76	0.82
Thalamus subcortical R	0.76	0.27
Vofa L	19.85	53.83
Vofa R	8.4	7.1
Vofb L	10.69	15.03
Vofb R	0.76	0.55

Supplement Table 4: Effect sizes for each DTI metric for each time point comparing Sham vs TBI rats for the left thalamic subcortical projections (Thalamus subcortical L) and left fimbria (Fimbria L).

DTI Metric	TP	Thalamus subcortical L (Cohen's D)	Fimbria L (Cohen's D)
AD	2d	0.68	0.61
AD	9d	0.12	-0.01
AD	1mo	0.34	-0.59
AD	5mo	-1.34	-1.92
FA	2d	0.4	0.61
FA	9d	0.09	0.62
FA	1mo	0.581	0.68
FA	5mo	-0.43	-0.49
MD	2d	0.69	0.4
MD	9d	-0.06	-0.5
MD	1mo	-0.7	-1.57
MD	5mo	-1.9	-2.61
RD	2d	0.34	-0.19
RD	9d	-0.25	-1.06
RD	1mo	-1.44	-2.27
RD	5mo	-1.71	-2.32

Supplement Table 5: Summary of Spearman's Correlation Analysis Between Apnea Severity and DTI Metrics for TBI rats. This table shows the correlation coefficients (R), unadjusted p-values, and FDR-corrected p-values for the associations between apnea severity and DTI metrics (AD, FA, MD, RD) within the left fimbria and the left thalamic subcortical projections across four time points post-injury (2 d, 9 d, 1 mo, 5 mo). Corrections for multiple comparisons were made using the Benjamini-Hochberg procedure to control the false discovery rate.

Metric	TP	Region	R	p-value	corrected p-value
AD	2d	Fimbria L	0.1300	0.2300	0.4377

Metric	TP	Region	R	p-value	corrected p-value
AD	2d	Thalamus subcortical L	0.0081	0.9403	0.9403
AD	9d	Fimbria L	0.1617	0.1418	0.3780
AD	9d	Thalamus subcortical L	-0.0485	0.6614	0.8733
AD	5mo	Fimbria L	0.2389	0.0553	0.1608
AD	5mo	Thalamus subcortical L	0.2700	0.0296	0.1054
AD	1mo	Fimbria L	0.3208	0.0035	0.0505
AD	1mo	Thalamus subcortical L	0.1213	0.2806	0.4988
FA	2d	Fimbria L	0.0234	0.8296	0.8733
FA	2d	Thalamus subcortical L	0.1081	0.3188	0.5370
FA	9d	Fimbria L	-0.0324	0.7702	0.8733
FA	9d	Thalamus subcortical L	0.1389	0.2077	0.4377
FA	5mo	Fimbria L	0.0370	0.7700	0.8733
FA	5mo	Thalamus subcortical L	0.1095	0.3854	0.6167
FA	1mo	Fimbria L	0.0597	0.5965	0.8299
FA	1mo	Thalamus subcortical L	0.0394	0.7269	0.8733
MD	2d	Fimbria L	0.1394	0.1977	0.4377
MD	2d	Thalamus subcortical L	-0.0421	0.6985	0.8733
MD	9d	Fimbria L	0.0627	0.5710	0.8299
MD	9d	Thalamus subcortical L	-0.2649	0.0149	0.0898
MD	5mo	Fimbria L	0.2828	0.0224	0.0898
MD	5mo	Thalamus subcortical L	0.4364	0.0003	0.0027 *
MD	1mo	Fimbria L	0.2904	0.0085	0.0683
MD	1mo	Thalamus subcortical L	0.0785	0.4862	0.7409
RD	2d	Fimbria L	0.2525	0.0183	0.0898
RD	2d	Thalamus subcortical L	0.0211	0.8460	0.8733
RD	9d	Fimbria L	0.1317	0.2326	0.4377
RD	9d	Thalamus subcortical L	-0.2194	0.0450	0.1439
RD	5mo	Fimbria L	0.1603	0.2022	0.4377
RD	5mo	Thalamus subcortical L	0.3462	0.0047	0.0505
RD	1mo	Fimbria L	0.2583	0.0199	0.0898
RD	1mo	Thalamus subcortical L	0.0292	0.7959	0.8733
